# Scalp Irradiation with 3D-Milled Bolus: Initial Dosimetric and Clinical Experience

**DOI:** 10.3390/cancers16040688

**Published:** 2024-02-06

**Authors:** Khaled Dibs, Emile Gogineni, Sachin M. Jhawar, Sujith Baliga, John C. Grecula, Darrion L. Mitchell, Joshua Palmer, Karl Haglund, Therese Youssef Andraos, Wesley Zoller, Ashlee Ewing, Marcelo Bonomi, Priyanka Bhateja, Gabriel Tinoco, David Liebner, James W. Rocco, Matthew Old, Mauricio E. Gamez, Arnab Chakravarti, David J. Konieczkowski, Dukagjin M. Blakaj

**Affiliations:** 1 Department of Radiation Oncology, The Ohio State University Wexner Medical Center, 460 W. 10th Ave., Columbus, OH 43210, USA; khaled.dibs2@osumc.edu (K.D.); emile.gogineni@osumc.edu (E.G.); sachin.jhawar@osumc.edu (S.M.J.); sujith.baliga@osumc.edu (S.B.); john.grecula@osumc.edu (J.C.G.); darrion.mitchell@osumc.edu (D.L.M.); joshua.palmer@osumc.edu (J.P.); karl.haglund@osumc.edu (K.H.); thereseyoussef.andraos@osumc.edu (T.Y.A.); wesley.zoller@osumc.edu (W.Z.); ashlee.ewing@osumc.edu (A.E.); arnab.chakravarti@osumc.edu (A.C.); david.konieczkowski@osumc.edu (D.J.K.); 2 Department of Medical Oncology, The Ohio State University Wexner Medical Center, 460 W. 10th Ave., Columbus, OH 43210, USA; marcelo.bonomi@osumc.edu (M.B.); priyanka.bhateja@osumc.edu (P.B.); gabriel.tinoco@osumc.edu (G.T.); david.liebner@osumc.edu (D.L.); 3 Department of Otolaryngology, The Ohio State University Wexner Medical Center, 460 W. 10th Ave., Columbus, OH 43210, USA; james.rocco@osumc.edu (J.W.R.); matthew.old@osumc.edu (M.O.); 4 Department of Radiation Oncology, Mayo Clinic, 200 First St. SW, Rochester, MN 55905, USA; gamezharo.mauricio@mayo.edu

**Keywords:** 3D-milled bolus, scalp, angiosarcoma, squamous cell carcinoma, radiotherapy, external beam, radiation, skin, cutaneous, VMAT, IMRT

## Abstract

**Simple Summary:**

Scalp irradiation for cutaneous malignancies presents complex challenges due to the high radiation dose, irregular surface anatomy, and radiosensitive organs at risk. Scalp convexity provides unique aspects to consider when attempting to deliver a homogenous dose with external beam radiation. Traditional bolus methods face several issues, including the risk of air gaps and placement difficulty due to skull shape. Here, we outline the steps involved with 3D-milled bolus creation, which has the potential to deliver a homogenous dose to the surface target with rapid dose falloff when treated with VMAT radiation. To our knowledge, this is the first study to report clinical outcomes using this approach. We showed homogenous dose target coverage and OAR doses lower than those reported in other studies utilizing alternative dose delivery and bolus techniques. We also showed that while acute skin toxicity was substantial, the treatment was well tolerated in later follow-up with only 5% late grade 3 toxicity.

**Abstract:**

Background and purpose: A bolus is required when treating scalp lesions with photon radiation therapy. Traditional bolus materials face several issues, including air gaps and setup difficulty due to irregular, convex scalp geometry. A 3D-milled bolus is custom-formed to match individual patient anatomy, allowing improved dose coverage and homogeneity. Here, we describe the creation process of a 3D-milled bolus and report the outcomes for patients with scalp malignancies treated with Volumetric Modulated Arc Therapy (VMAT) utilizing a 3D-milled bolus. Materials and methods: Twenty-two patients treated from 2016 to 2022 using a 3D-milled bolus and VMAT were included. Histologies included squamous cell carcinoma (*n* = 14, 64%) and angiosarcoma (*n* = 8, 36%). A total of 7 (32%) patients were treated in the intact and 15 (68%) in the postoperative setting. The median prescription dose was 66.0 Gy (range: 60.0–69.96). Results: The target included the entire scalp for 8 (36%) patients; in the remaining 14 (64%), the median ratio of planning target volume to scalp volume was 35% (range: 25–90%). The median dose homogeneity index was 1.07 (range: 1.03–1.15). Six (27%) patients experienced acute grade 3 dermatitis and one (5%) patient experienced late grade 3 skin ulceration. With a median follow-up of 21.4 months (range: 4.0–75.4), the 18-month rates of locoregional control and overall survival were 75% and 79%, respectively. Conclusions: To our knowledge, this is the first study to report the clinical outcomes for patients with scalp malignancies treated with the combination of VMAT and a 3D-milled bolus. This technique resulted in favorable clinical outcomes and an acceptable toxicity profile in comparison with historic controls and warrants further investigation in a larger prospective study.

## 1. Introduction

Cutaneous malignancies of the head and neck region represent a complex group of cancers due to their poor prognosis, treatment complexity, and risk of treatment-related toxicity. The National Comprehensive Cancer Network (NCCN) guidelines list the scalp as a high-risk region for cutaneous squamous cell carcinoma (SCC) [1], with multiple studies providing corroborating evidence [2,3,4,5,6]. Cutaneous angiosarcomas are rare tumors of vascular endothelial cells with an aggressive natural history [7]. The majority of cutaneous SCC and angiosarcoma occur on the face and scalp [8,9,10].

Surgical resection remains the standard-of-care treatment for both cutaneous SCC and angiosarcoma [1,11]. Multidisciplinary evaluation is imperative, as surgery in this region can be associated with significant morbidity depending on the location and extent of the disease, and achieving widely negative margins is often challenging. Radiation therapy (RT) is often indicated, either in the adjuvant setting after surgery or definitively in patients not undergoing surgery [4,6,12,13,14,15].

The delivery of RT to SCC and angiosarcoma on the face and scalp presents complex challenges due to the high doses of radiation required, irregular surface anatomy, and radiosensitive organs at risk (OAR) in close proximity, such as the brain, optic structures, and lacrimal glands. Scalp RT can be delivered via brachytherapy and external beam RT using electrons, protons, and photons with intensity-modulated RT (IMRT) and Volumetric-Modulated Arc Therapy (VMAT) [16,17,18,19,20,21,22,23,24]. The convexity of the scalp provides unique aspects to consider when attempting to deliver a uniform RT dose. This requires reproducible setup and plan delivery, the consideration of RT technique and beam angles, and minimizing air gaps if utilizing a bolus to increase skin dose. Over the last three decades, technical advancements in scalp treatment, from electron–photon field matching to VMAT, have improved dose homogeneity [12,15,16,17]. VMAT can eliminate many of the concerns associated with traditional match-based techniques, such as shifting and field matching requirements. Additionally, inverse planning optimization can allow for simultaneous integrated boosting to high-risk areas in a single plan, eliminating the need to boost sequentially with electron fields.

A bolus is required when treating cutaneous lesions with photons in order to provide an adequate surface dose. Commonly employed forms of boluses include commercial flat and three-dimensional (3D)-printed boluses. A commercial flat bolus faces several issues, including a significant risk of air gaps and placement difficulty due to skull shape and gravity when treating patients in a supine position [25]. A 3D-printed bolus improves dose homogeneity, dose distribution, and conformity index in conjunction with VMAT [26,27]. However, multiple concerns persist even with a 3D-printed bolus, such as air gaps secondary to layer resolution, fill space, print strength, and print pattern limitations [28]. Additionally, the interior surfaces of a 3D-printed bolus may not be rendered smooth, which can have negative impacts on patient comfort and dose homogeneity. Finally, the density and rigidity of a 3D-printed bolus can vary based on the type of filament used in its creation.

The 3D-milled bolus method may mitigate many of the aforementioned issues that face treatment with commercial flat and 3D-printed boluses. Unlike a 3D-printed bolus, a 3D-milled bolus is formed by a subtractive Computer Numerical Control (CNC) milling process from a uniform-density block, resulting in a final 1 cm custom bolus that allows improved dose coverage and homogeneity [28]. Here, we describe the process of 3D-milled bolus creation and evaluate dosimetry, toxicity, control, and survival for patients with scalp SCC and angiosarcoma treated with VMAT using a 3D-milled bolus.

## 2. Materials and Methods

### 2.1. Study Design

We conducted an Institutional Review Board-approved retrospective analysis on patients with malignant disease of the scalp who received external beam RT using 3D-milled bolus and VMAT technique at our National Cancer Institute-Designated Cancer Center from 2016 to 2022. Patients were initially staged based on the American Joint Committee on Cancer (AJCC) edition that was current at the time of diagnosis but were retrospectively staged using the AJCC eighth edition for the purpose of this analysis.

### 2.2. CT Simulation and 3D-Milled Bolus Formation

Creation of the 3D-milled bolus involves two phases [28]. In phase 1, patients underwent computed tomography (CT) simulation in the supine position with a clear plastic Silverman headrest. Radiopaque wires were placed by the attending physician to identify the postoperative scar and/or gross disease and other at-risk areas to be included in the target volumes, as well as the whole scalp. Patients planned for treatment of the scalp and neck were scanned from the scalp vertex superiorly to the diaphragm inferiorly. For patients planned for treatment of the scalp alone, scan length was limited to the wired region with enough margin to allow creation of bolus in the treatment planning system (TPS).

**Figure 1:**
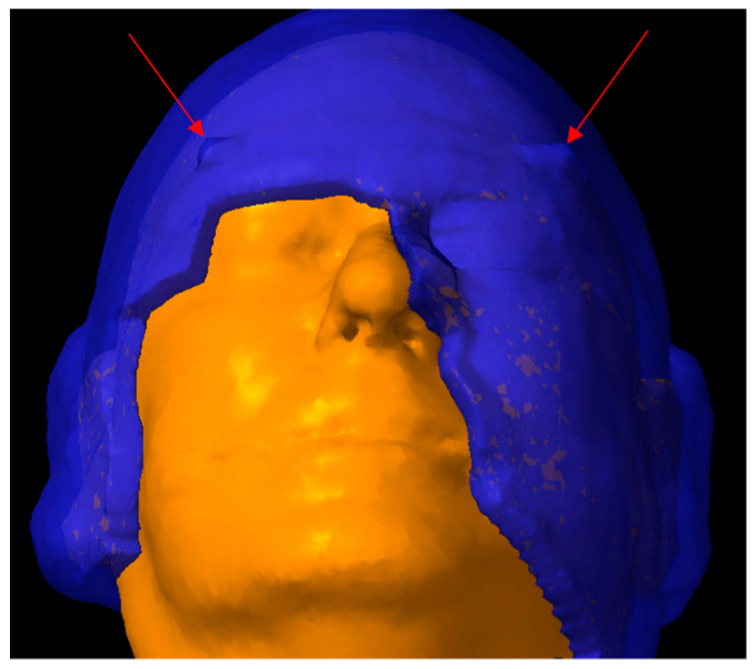
The red arrows indicate the two indexing “horns”, which are manually added onto the anterolateral aspect of the virtual bolus contour. These horns limit the rotation of the bolus under the thermoplastic mask that is created during phase 2 of 3D-milled bolus formation.

A 3D-virtual bolus was generated in the Eclipse TPS (Varian, Palo Alto, CA, USA). The external body contour was created in an automated fashion via TPS and reviewed by the assigned medical dosimetrist and attending physician. This often involved manual editing, particularly in areas with irregular surface structures, such as the earlobes and eyebrows. The radiopaque wires were converted to high-resolution structures. A rind structure was created by expanding the external contour outward 1 cm uniformly. An additional volume was generated using a 2 cm outward expansion uniformly from the outermost wire to give bolus margin from the field edge. Finally, a bolus contour was created using overlap of these two volumes. As shown in Figure 1, two indexing “horns” were manually added onto the anterolateral aspect of the virtual bolus contour to limit rotation of the bolus under the thermoplastic mask that was created during phase 2 of this process.

This 3D virtual bolus representation was reviewed by the attending physician and edited when appropriate, such as the removal of thin areas around the eyebrow and post-auricular surface. The virtual bolus structure was then delivered in digital imaging and communication in medicine (DICOM) format to a third-party vendor to be milled from a proprietary wax compound with an electron density of 0.92 g/cm^3^.

**Figure 2:**
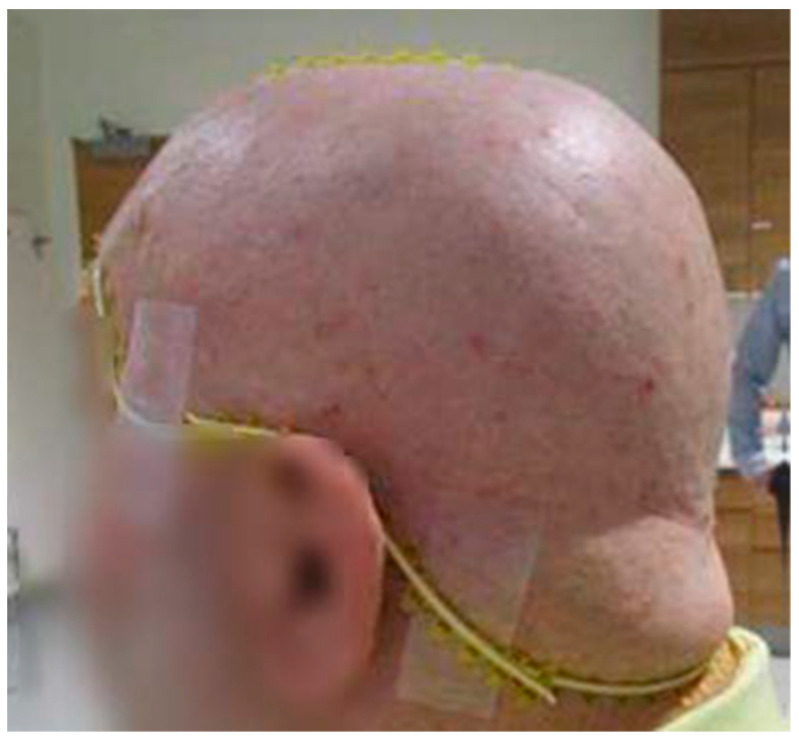
The 3D-milled bolus delivered to the radiation oncology department. This was placed on the patient’s scalp, and the treatment area was outlined using a radiopaque wire to aid in target delineation and bolus fabrication.

**Figure 3:**
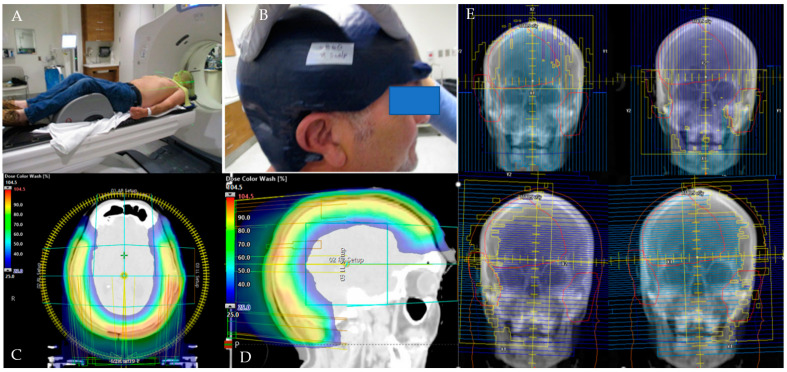
Shown here is the CT simulation (**A**), the creation of the 3D-milled bolus (**B**), dose-color-wash images in the axial (**C**) and sagittal (**D**) planes, and collimator arrangements (**E**) for a 60-year-old immune-compromised patient with a diagnosis of pT3N0 cM0 Stage III cutaneous squamous cell carcinoma of the scalp. He underwent surgical resection but experienced rapid recurrence with a skip lesion away from the operative bed. He was treated with concurrent chemoradiation to a dose of 69.96 Gy to the gross disease (yellow), 66.0 Gy to the postoperative surgical bed (green), and 59.4 Gy to the remaining portion of the scalp (cyan), all in 33 fractions via simultaneous integrated boost. As shown, the 3D-milled bolus (**B**) and VMAT planning techniques allowed for adequate target coverage at the surface and rapid dose fall off at depth within the brain (**C**,**D**). The patient experienced acute grade 1 fatigue, grade 2 pain, grade 3 dermatitis, and hair loss during treatment but no late toxicity. At the time of analysis, the patient was alive without recurrence 29 months post-treatment.

Phase 2 began after 3D-milled bolus was created and returned to the radiation oncology department, as shown in Figure 2 and Figure 3.

Patients underwent a second CT simulation in a similar position to phase 1 but with the 3D-milled bolus in place on their scalp and a MOLDCARE custom headrest holding the bolus in place, conforming to its posterior aspect. A custom thermoplastic mask was created covering the patient’s head and shoulders, during which the aforementioned index points on the bolus were registered into the mask to ensure a reproducible setup. A rigid fusion was performed between the two CT simulation scans, allowing for the radiopaque wires to delineate the high-risk volume on the planning CT.

### 2.3. Target Delineation and Treatment Planning

Target delineation and treatment planning were conducted in Eclipse TPS. Target volumes included either the partial or entire scalp. For those receiving partial scalp radiation only, a minimum of 1 cm margin was added beyond the gross tumor in the clinical target volume. Then, 0.3–0.5 cm margins in all directions were added for planning target volumes (PTVs), cropped at the skin surface. For patients with unresected disease or positive surgical margins, an appropriate boost volume was delineated. Patients with gross nodal disease and those deemed to be at risk for subclinical nodal spread also received neck nodal irradiation. All patients were planned using VMAT techniques with 6 MV photon beams, utilizing VMAT with daily Cone-Beam CT and 6 degrees of freedom couch tops. For total scalp volumes, four full coplanar VMAT arcs were used. Collimation was offset directionally in each of the four quadrants (left, right, superior, and inferior) to promote increased midline structure sparing with multi-leaf collimation (MLC). Simultaneous integrated boost techniques with dose painting were used for patients with multiple target volumes, such as those receiving a boost to gross disease and/or those receiving elective neck RT. Dose homogeneity index (DHI) was calculated using the equation DHI = D_5%_/D_95%_, with a DHI of 1.0 representing an ideal value [29].

### 2.4. Study Endpoints

Patient demographics, tumor characteristics, dose volume histograms, clinical outcomes, and toxicity were recorded. Immune-compromised patients were defined as those with a history of organ transplant, lymphoma, chronic lymphocytic leukemia, drug-induced immunosuppression, and human immunodeficiency virus infection [30,31,32,33,34,35]. Toxicity was classified according to the National Cancer Institute Common Terminology Criteria for Adverse Events version 5.0 (CTCAE v5.0). Locoregional control (LRC) was defined as disease control within the scalp and neck, and distant metastasis-free survival (DMFS) referred to control outside of the head and neck.

### 2.5. Patient Characteristics

Patient, oncologic, and treatment details are provided in Table 1.

Twenty-two patients were included for analysis, with a median follow-up of 21.4 months (range, 4.0–75.4). Median age was 74 (range, 46–85), and ECOG performance status was 0–1 in 19 (86%) patients. Eight (36%) patients were immune-compromised, including four patients with organ transplants (two with lung transplants, one with heart transplant, and one with renal/pancreas transplant), two patients with diffuse large B-cell lymphoma, one with chronic lymphocytic leukemia, and one with a history of acute myeloid leukemia for which they underwent bone marrow transplant. Histologies included SCC in 14 (64%) patients and angiosarcoma in 8 (36%) patients. A total of 17 (77%) patients had T3–4 disease, and 19 (86%) were N0. A total of seven (32%) patients were treated with definitive intent to unresected gross disease. Of the remaining 15 (68%) treated postoperatively, 5 (33%) had microscopically positive margins (the remainder being microscopically negative), 4 (27%) had perineural invasion (PNI), and none had lymphovascular invasion (LVI). No patient underwent cervical lymph node dissection. Median prescription dose was 66.0 Gy (range, 60.0–69.96) in a median of 33 fractions (range, 30–33). A total of nine (41%) patients received systemic therapy concurrent with radiation, including chemotherapy in five (23%) and immunotherapy in four (19%).

### 2.6. Statistical Analysis

Statistical analyses were conducted using SPSS Statistics version 28 (Armonk, NY, USA). Continuous patient demographics were described using medians and ranges. Frequency counts and proportions were used to describe discrete variables. Kaplan–Meier curves were used to assess LRC, DMFS, overall survival (OS), and the relationship between baseline and treatment characteristics with control and survival. Independent-sample Mann–Whitney U tests were used to investigate the correlation between continuous variables. Independent-sample *t*-tests were performed to examine the correlation between continuous and dichotomized variables.

## 3. Results

Details regarding dose fractionation, target volumes, and dosimetric variables are provided in Table 2.

The target included the entire scalp for 8 (36%) patients; in the remaining 14 (64%), the median ratio of PTV to scalp volume was 35% (range, 25–90%). A total of five (23%) patients received neck irradiation, including two cN0 patients who were treated with elective ipsilateral nodal RT to 54.12 Gy and three with gross nodal involvement (two received 59.4 Gy to the involved neck, and one received 59.4 Gy to the involved neck and 54.12 Gy to the contralateral uninvolved neck). All gross nodal diseases received full prescription doses (two patients received 66.0 Gy, and one patient received 69.96 Gy). Ten (45%) patients had a single PTV, which was treated to 60.0 Gy (*n* = 9) or 66.0 Gy (*n* = 1). Nine (41%) patients had two PTVs, which were treated to 54.0 and 60.0 Gy (*n* = 1) or 59.4 and 66.0 Gy (*n* = 8). Two (9%) patients had three PTVs, which were treated to 54.12, 59.4, and 69.96 Gy (*n* = 1) or 59.4, 66.0, and 69.96 Gy (*n* = 1). One (5%) patient had four PTVs, which were treated to 54.12, 59.4, 66.0, and 69.96 Gy.

The median brain maximum and mean doses were 65.0 Gy (range, 52.5–71.9) and 13.0 Gy (range, 1.4–38.4), respectively. Patients treated with partial scalp irradiation had significantly lower mean brain doses than those with whole scalp RT (10.2 Gy vs. 23.6 Gy, *p* = 0.001). The median hippocampus Dmin and Dmax were 5.7 Gy (range, 0.1–14.9) and 10.0 Gy (range, 0.3–23.0), respectively. The median DHI was 1.07 (range, 1.03–1.15). The median PTV Dmax dose was 72.3 Gy (range, 66.2–79.6), and the median PTV Dmax percentage was 111.3% (range, 105.0–118.0).

Acute and late toxicity rates are provided in Table 3.

The most common acute side effects were grade 1–2 fatigue in 22 (100%) patients, grade 1–2 pain in 17 (78%) patients, and grade 1–2 dermatitis in 16 (73%) patients. Six (27%) patients experienced acute grade 3 dermatitis. Late side effects included four (18%) patients with grade 1 memory impairment and grade 1 eye dryness, one (5%) patient with grade 2 osteoradionecrosis and grade 2 skin ulceration, and one (5%) patient with grade 3 dermatitis. No patient developed brain radionecrosis. No factors were found to be significantly associated with toxicity, including prescription dose, use of systemic therapy, and all analyzed dosimetric parameters.

Table 4 summarizes the rates of control and survival, in addition to univariate analyses of variables associated with each.

Curves for LRC, DMFS, and OS are shown in Supplementary Figures S1–S3. Furthermore, the 18-month rates of LRC, DMFS, and OS were 75%, 62%, and 79%, respectively. Median LRC, DMFS, and OS for all patients were 20.0 (range, 2.0–75.0), 15.6 (range, 4.0–75.4), and 21.4 months (range, 4.0–75.4), respectively.

Supplementary Table S1 provides additional information on each of the five locoregional recurrences. As shown in Supplementary Figure S1B,C, immune status and N-stage were significantly associated with LRC (immune compromised vs. immune competent: 42% vs. 92% at 18 months, *p* = 0.006; N0 vs. N ≥ 1: 82% vs. 34% at 18 months, *p* = 0.028). As shown in Supplementary Figure S2B,C, variables significantly associated with DMFS included N-stage (N0 vs. N ≥ 1: 90% vs. 0% at 18 months, *p* = 0.011) and treatment context (intact/unresected disease vs. adjuvant/postoperative: 24% vs. 77% at 18 months, *p* = 0.020). As shown in Supplementary Figure S3B, the only factor significantly associated with OS was N-stage (N0 vs. N ≥ 1: 85% vs. 34% at 18 months, *p* < 0.001).

## 4. Discussion

This represents the first study, to our knowledge, reporting clinical outcomes for patients with cutaneous SCC and angiosarcoma of the scalp treated with the combination of VMAT and a 3D-milled bolus. We made the following observations: (1) a 3D-milled bolus allowed for homogenous dose coverage with VMAT, with a median dose homogeneity index of 1.07; (2) while acute toxicity was substantial with 27% acute grade 3 dermatitis, the treatment was well tolerated in later follow-up, with only 5% late grade 3 toxicity; (3) this approach provided high rates of control and survival; and (4) node-positive disease was significantly associated with worse LRC, DMFS, and OS, and the rates of LRC were lower in immune-compromised patients.

The conformality and reproducibility of the 3D-milled bolus in combination with advanced VMAT planning techniques allowed robust PTV coverage and sparing of surrounding tissue. Doses to OARs were acceptably low using this technique in comparison to outcomes from studies evaluating alternative methods of planning and bolus. Rakici et al. compared five different plans when treating with whole scalp irradiation to 66.0 Gy (the median dose in our cohort) using VMAT, with each plan utilizing different planning and bolus techniques [26]. Maximum brain doses were between 66.6 and 71.5 Gy, all higher than the median of our cohort of 65.0 Gy. Kai et al. conducted a dosimetric study comparing VMAT, 5-field IMRT, and 9-field IMRT plans with a 1 cm bolus when treating angiosarcoma of the scalp to 60.0 Gy [16]. Mean brain doses and maximum eye doses were 18.3–23.5 and 18.0–24.7 Gy, respectively. Hu et al. compared 3D-conformal, IMRT, and various RapidArc plans for scalp irradiation using a 1 cm bolus when treating with photons to 70.0 Gy. Mean brain and maximum eye doses were 19.2–30.0 and 27.9–49.1 Gy, respectively [36]. Each plan from both of these studies resulted in higher doses to OARs than the outcomes from our cohort, in which the mean brain and maximum eye doses were 13.0 and 10.0 Gy, respectively.

The rate of late radiation dermatitis was relatively low, with 5% of patients experiencing late grade 3 skin ulceration; however, the 27% rate of grade 3 acute dermatitis highlights the importance of aggressive skin and wound care during treatment and in short-term follow-up. Our institution places a high priority on proactive evaluation and treatment by physicians and nursing teams for all patients with scalp malignancies treated with external beam RT, including the early involvement of specialized wound care nurses. There were no tissue changes related to inflammation or swelling that caused issues with the fit of the bolus and mask during treatment for any patient. For patients who experienced scalp pain secondary to dermatitis during treatment, topical lidocaine was often employed in addition to oral pain medication when indicated.

The control and survival rates in our cohort of patients were similar or higher than other retrospective studies reporting outcomes for SCC and angiosarcoma. Pawlik et al. published a study of 29 patients with angiosarcoma of the scalp treated at the University of Michigan with surgery, 79% of whom received adjuvant RT [15]. At a median follow-up of 18.2 months, they showed LRC and OS rates of 27.6% and 41.4%, respectively. Patel et al. reported on a similar cohort of 55 patients with angiosarcoma of the face or scalp treated at The Mayo Clinic, the majority of whom received surgery and adjuvant radiation [14]. At a median follow-up of 25.2 months, they showed a median survival of 25.2 months and 5-year LRC and OS of 18% and 38%, respectively. While only eight patients with angiosarcoma were included in our analysis, the LRC and OS rates at 18 months were both 100%, with a median of 24.8 months for both LRC and OS. While our 18-month rates of LRC and OS for SCC of 60–66% are lower than those of low-risk cutaneous SCC, several factors that were prevalent in our cohort are shown to be highly correlative with poorer outcomes, including immunosuppression, positive margins, and lymph node metastases [6].

Our results compared favorably to other studies in which patients with scalp tumors were treated with a bolus and external beam radiation with regard to dosimetric, oncologic, and toxicity outcomes. As discussed above, doses to the brain (maximum dose: median, 65.0 Gy; range, 52.5–71.9; mean dose: median, 13.0 Gy; range, 1.4–38.4) and eyes (maximum dose: median, 10.0 Gy; range, 0.2–49.0) were lower in our cohort than those reported in the literature [16,26,36]. The rates of control (18-month LRC 75%) and survival (18-month OS 79%) were similar or higher in our cohort than those reported in the literature [15,16]. Given the relatively small number of patients and heterogenous characteristics such as immune-compromised status, histology, stage, and target volumes, caution should be taken when interpreting clinical outcomes and when comparing results to other published studies. Similarly, the rates of toxicity (10% rate of late grade ≥2 toxicity) were lower than those reported in other studies, such as those by Guadagnolo et al., which showed an actuarial rate of 47% late RT-induced complications [12].

When utilizing this method of a 3D-milled bolus in combination with VMAT, one must consider the cost of purchase, potential delays, and risks, such as progression while awaiting bolus creation and flap swelling causing issues with reproducible fit in postoperative patients. Bolus pricing depends on treatment volume, with the price increasing proportionately to the required size of the bolus. When targets include the entire scalp, we order the largest available size, while smaller bolus sizes may be used for smaller targets. When using a traditional bolus, we tend to rely on Elasto-Gel sheets, which typically require at least 2–3 modified sheets to cover the area encompassed by one 3D-milled bolus, such as an entire scalp. On average, the price of a 3D-milled bolus is around 2.5 times higher than that of traditional bolus. Excluding processing and shipping, manufacture requires a minimum of 8 h from the third-party vendor and can take up to 2 business days. The time required to produce this 3D-milled bolus and, correspondingly, the potential delay of treatment initiation exceeds that of a traditional bolus, which can be created on the same day of simulation and rarely would require more than an hour of time for production. Outside of patients requiring urgent treatment, our routine time from simulation to treatment is around 2 weeks, and thus, the process of 3D-milled bolus creation does not usually cause delays in treatment initiation. We would not recommend the use of this form of bolus for patients who require urgent start to treatment, such as those with symptoms requiring palliation, patients with intact bulky disease, and those who have already experienced significant delays after surgery. Of the 22 patients included in this study, no patient experienced tumor growth or progression while awaiting bolus creation, nor did postoperative edema or swelling cause issues with a reproducible setup of the 3D-milled bolus. Nevertheless, we routinely account for this risk by ordering a small expansion in case we have some small changes or errors in our surface contour. Additionally, this product is pliable and allows some room to adapt to the new contour, so small changes in growth or swelling do not adversely impact reproducible setup and fit.

This study is limited by its retrospective design and, therefore, inherent biases that affect all retrospective studies, such as recall bias, misclassification bias, confounding variables, and the selection bias of who was treated with whole scalp versus partial scalp RT. Correspondingly, the results from this study cannot determine causation, only association. Given that we did not perform matched cohort comparisons, definitive conclusions cannot be made on the superiority of this technique over other planning and bolus methods. Another limitation is the small sample size, and thus, strong conclusions should not be drawn for control and survival outcomes for each histology individually. Additional retrospective studies with larger patient cohorts and prospective evidence are paramount to better understanding outcomes using this approach to treatment.

## 5. Conclusions

Our study showed that the combination of VMAT planning with a 3D-milled bolus allows for safe and effective radiotherapy for squamous cell carcinoma and angiosarcoma of the scalp, resulting in promising clinical outcomes and an acceptable toxicity profile. This approach warrants further investigation in a larger prospective study.

## Figures and Tables

**Table 1:** Patient clinical, oncologic, and treatment-related information.

Variable (*n* = 22)				*n* (%)
Follow-up (mo): median (range)				21.4 (4.0–75.4)
Age (yr): median (range)				74 (46–85)
Sex				
	Male			20 (91)
	Female			2 (9)
ECOG performance status				
	0			16 (73)
	1			3 (14)
	2			3 (14)
Immune compromised				
	Yes			8 (36)
	No			14 (64)
Histopathology				
	Squamous cell carcinoma			14 (64)
	Angiosarcoma			8 (36)
Stage				
	Squamous cell carcinoma			
		T-stage		
			T1	0 (0)
			T2	1 (7)
			T3	8 (57)
			T4a	5 (36)
		N-stage		
			N0	12 (86)
			N1	0 (0)
			N2	2 (14)
	Angiosarcoma			
		T-stage		
			T1	0 (0)
			T2	4 (50)
			T3	3 (37)
			T4a	1 (13)
		N-stage		
			N0	7 (87)
			N1	1 (13)
			N2	N/A
Concurrent systemic therapy				
	None			13 (59)
	Paclitaxel			5 (23)
	Cemiplimab			3 (14)
	Nivolumab			1 (5)

Abbreviations: mo—months; yr (years); ECOG—Eastern Cooperative Oncology Group; R0—negative margins; R1—microscopically positive margins; PNI—perineural invasion; LVI—lymphovascular invasion.

**Table 2:** Radiation treatment planning and dosimetric variables.

Variable		Median (Range)
Extent of radiation: *n* (%)		
	Whole scalp	8 (36)
	Partial scalp	14 (64)
	Scalp + neck	5 (23)
Prescription dose (Gy)		66.0 (60.0–69.96)
	PTV54.0–54.12 (*n* = 3) (cc)	538 (121–624)
	PTV59.4–60.0 (*n* = 21) (cc)	355 (40–1352)
	PTV66.0 (*n* = 11) (cc)	130 (62–789)
	PTV69.96 (*n* = 3) (cc)	107 (6–827)
	Gross disease (Gy)	66.0 (66.0–69.96)
	Adjuvant (Gy)	66.0 (60.0–66.0)
Number of fractions: *n* (%)		
	30 fractions	10 (45)
	33 fractions	12 (55)
Skin V60 (cc)		268 (19–598)
Skin V64 (cc)		112 (1–580)
Brain Dmax * (Gy)		65.0 (52.5–71.9)
Brain Dmean (Gy)		13.0 (1.4–38.4)
Eye Dmax * (Gy)		10.0 (0.2–49.0)
Cochlea Dmean (Gy)		8.0 (0.1–20.4)
Lacrimal gland Dmean (Gy)		6.4 (0.1–38.0)
Hippocampus Dmin (Gy)		5.7 (0.1–14.9)
Hippocampus Dmax * (Gy)		10.0 (0.3–23.0)
PTV Dmax *		
	Dose (Gy)	72.3 (66.2–79.6)
	Percentage (%)	111.3 (105.0–118.0)
Dose homogeneity index		1.07 (1.03–1.15)
	D2% (Gy)	70.3 (63.4–76.9)
	D98% (Gy)	65.1 (57.6–69.5)

Abbreviations: PTV—planning target volume; * maximum dose to 0.03 cc.

**Table 3:** Acute and late toxicity classified according to CTCAE v5.0.

Toxicity		*n* (%)
Acute side effects		
Fatigue		
	Grade 0	0 (0)
	Grade 1	18 (82)
	Grade 2	4 (18)
Pain		
	Grade 0	5 (23)
	Grade 1	14 (64)
	Grade 2	3 (14)
Dysgeusia		
	Grade 0	17 (77)
	Grade 1	5 (23)
Dermatitis		
	Grade 0	0 (0)
	Grade 1	5 (23)
	Grade 2	11 (50)
	Grade 3	6 (27)
Late side effects		
Osteoradionecrosis		
	Grade 0	21 (95)
	Grade 1	0 (0)
	Grade 2	1 (5)
Skin ulceration		
	Grade 0	20 (90)
	Grade 1	0 (0)
	Grade 2	1 (5)
	Grade 3	1 (5)
Memory impairment		
	Grade 0	18 (82)
	Grade 1	4 (18)
Eye dryness		
	Grade 0	18 (82)
	Grade 1	4 (18)

Abbreviations: CTCAE v5.0—National Cancer Institute Common Terminology Criteria for Adverse Events version 5.0.

**Table 4:** Univariate analysis of factors associated with rates of locoregional control, distant metastasis-free survival, and overall survival. Outcomes reported at 18 months.

Variable (*n* = 22)		LRC (%)	*p*-Value	DMFS (%)	*p*-Value	OS (%)	*p*-Value
Overall cohort		75		62		79	
ECOG performance status			0.647		0.656		0.269
	0	72		62		86	
	1	100		50		50	
	2	67		66		66	
Immune compromised			0.006 *		0.527		0.106
	Yes	42		56		56	
	No	92		67		92	
Histopathology			0.058		0.781		0.397
	Squamous cell carcinoma	60		66		66	
	Angiosarcoma	100		57		100	
T-stage			0.780		0.921		0.791
	T2	80		80		100	
	T3	70		62		75	
	T4a	83		52		62	
N-stage			0.028 *		<0.001 *		<0.001 *
	N0	82		74		85	
	N ≥ 1	34		0		34	
Treatment intent			0.560		0.020 *		0.092
	Intact/unresected disease	72		24		74	
	Adjuvant/postoperative	78		77		84	
Surgical margin			0.971		0.297		0.089
	Negative	75		88		100	
	Positive	80		60		60	
PNI			0.884		0.321		0.557
	Negative	75		60		82	
	Positive	75		75		75	
Systemic therapy			0.970		0.057		0.060
	Yes	78		39		64	
	No	72		81		90	

Abbreviations: LRC—locoregional control; DMFS—distant metastasis-free survival; OS—overall survival; ECOG—Eastern Cooperative Oncology Group; PNI—perineural invasion. * Considered statistically significant based on *p*-value < 0.050.

## Data Availability

Research data are stored in an institutional repository and will be shared upon request to the corresponding author.

## Supplementary Materials

The following supporting information can be downloaded at: https://www.mdpi.com/article/10.3390/cancers16040688/s1, Figure S1: Locoregional control (LRC) for the entire cohort (S1A) and separated based on immune status (S1B) and N-stage (S1C). The 18-month LRC was 75% with a median LRC of 20.0 months (range, 2.0–75.0). The 18-month LRC was significantly lower for immune-compromised patients than for immune-competent patients (42% vs. 92%, *p* = 0.006) and patients with N ≥ 1 versus N0 disease (34% vs. 82%, *p* = 0.028). Figure S2: Distant metastasis-free survival (DMFS) for the entire cohort (S2A) and separated based on N-stage (S2B) and surgical resection (S2C). The 18-month DMFS was 62% with a median DMFS of 15.6 months (range, 4.0–75.4). The 18-month DMFS was significantly lower for patients with N ≥ 1 versus N0 disease (0% vs. 90%, *p* = 0.011) and patients treated with definitive radiation versus those who underwent surgical resection (24% vs. 77%, *p* = 0.020). Figure S3: Overall survival (OS) for the entire cohort (S3A) and separated based on N-stage (S3B). The 18-month OS was 79% with a median OS of 21.4 months (range, 4.0–75.4). The 18-month OS was significantly lower for patients with N ≥ 1 versus N0 disease (34% vs. 85%, *p* < 0.001). Table S1: Clinical history, treatment information, nature of recurrence, and status for all patients who experienced locoregional failure.
