# Supplementary material for: Scalp Irradiation with 3D-Milled Bolus: Initial Dosimetric and Clinical Experience
Source: Cancers (Basel). 2024 Feb 6;16(4):688. doi: 10.3390/cancers16040688 (PMC10887235; doi:10.3390/cancers16040688)
Supplement: Supplementary file 1 [file cancers-16-00688-s001.zip › cancers-2839877-supplementary.pdf]

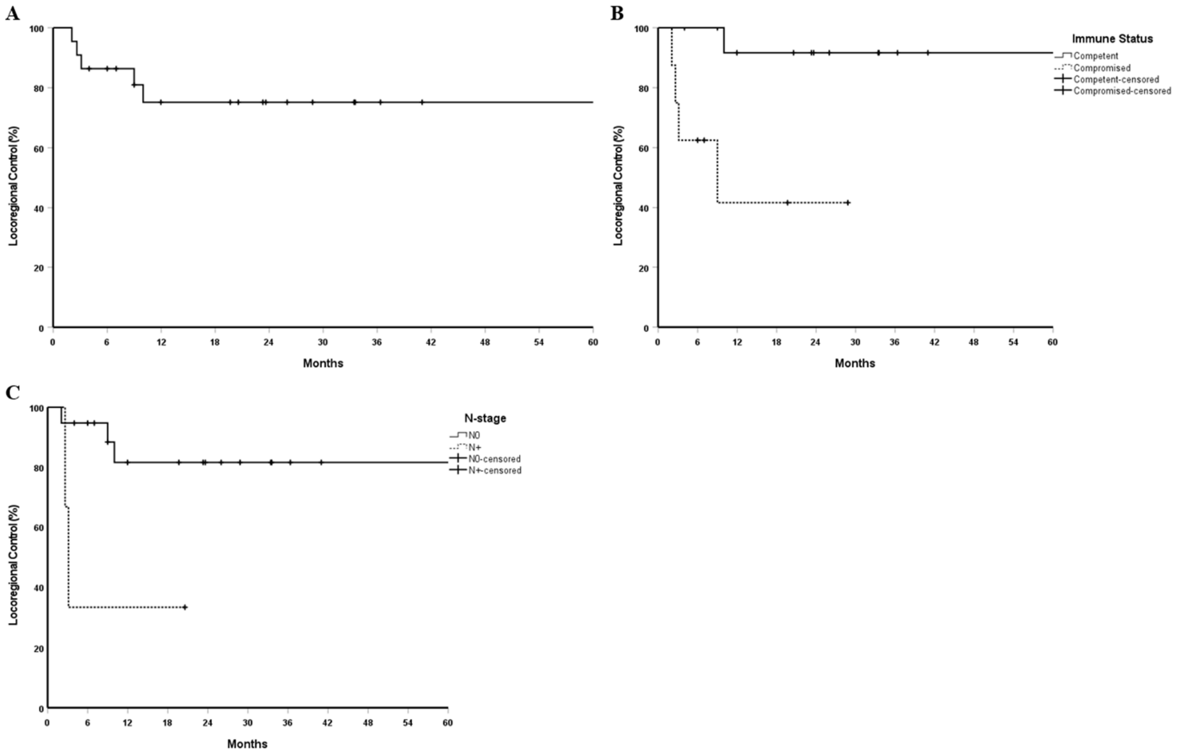

Figure S1. (A–C) Locoregional control (LRC) for the entire cohort (S1A) and separated based on immune status (S1B) and N-stage (S1C). 18-month LRC was 75% with median LRC of 20.0 months (range, 2.0-75.0). 18-month LRC was significantly lower for immune compromised patients than for immune competent patients (42% vs. 92%, p = 0.006) and for patients with N≥1 versus N0 disease (34% vs. 82%, p = 0.028).

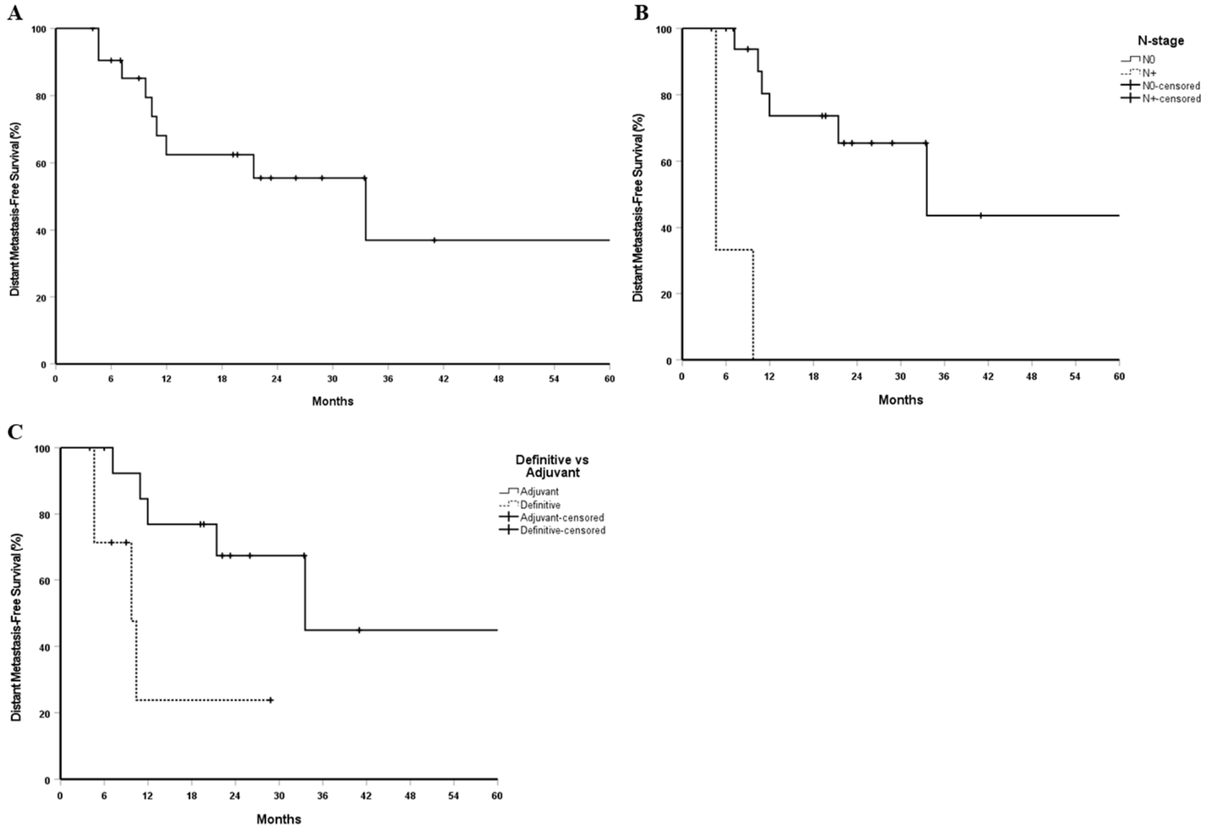

Figure S2: (A–C) Distant metastasis-free survival (DMFS) for the entire cohort (S2A) and separated based on N-stage (S2B) and surgical resection (S2C). 18-month DMFS was 62% with median DMFS of 15.6 months (range, 4.0-75.4). 18-month DMFS was significantly lower for patients with N⩾1 versus N0 disease (0% vs. 90%, p = 0.011) and for patients treated with definitive radiation versus those who underwent surgical resection (24% vs 77%, p = 0.020).

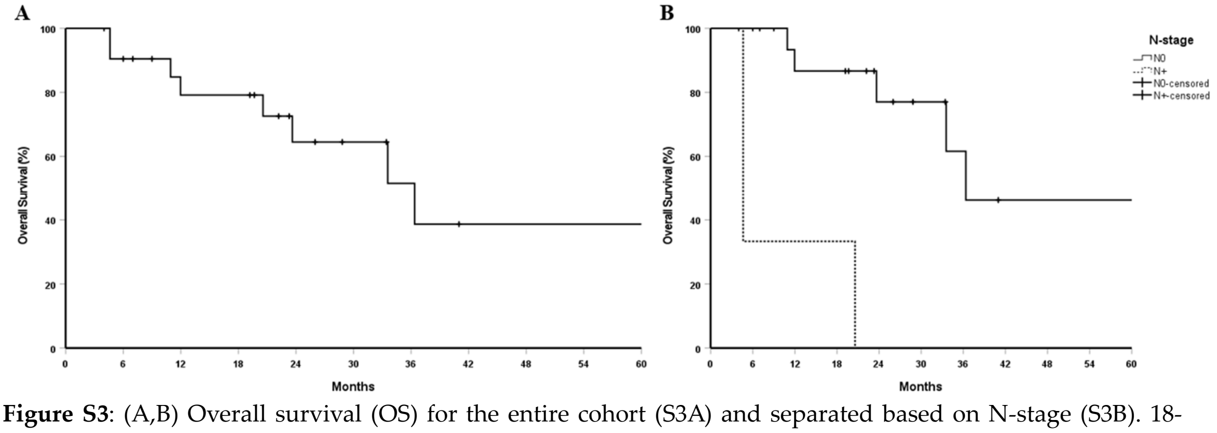

Figure S3: (A,B) Overall survival (OS) for the entire cohort (S3A) and separated based on N-stage (S3B). 18-month OS was 79% with median OS of 21.4 months (range, 4.0-75.4). 18-month OS was significantly lower for patients with N⩾1 versus N0 disease (34% vs. 85%, p < .001).

Table S1. Clinical history, treatment information, nature of recurrence, and status for all patients who experienced locoregional failure.

History	Radiation dose, fractionation, and target	Nature of recurrence	Status at the time of publication
61-year-old immune compromised man with cT4aN2b SCC treated with definitive radiation and concurrent nivolumab	69.96 Gy to gross disease in primary and lymph node, 59.4 Gy to the scalp, and 54.12 Gy to bilateral neck in 33 fractions	In-field; three months post-treatment	Died 5 months post-treatment
72-year-old immune compromised man with pT3N0 SCC treated with surgery with a positive margin and positive PNI	66 Gy in 33 fractions to the tumor bed	Marginal on scalp along V1; two months post-treatment	Died 11 months post-treatment
68-year-old immune compromised man with pT3N0 SCC treated with surgery with negative margins and negative PNI	60 Gy in 30 fractions to the tumor bed	Untreated portion of scalp along V1; 9 months post-treatment	Alive 19 months post-treatment
80-year-old immune competent man with pT2N0 SCC treated surgery with negative margins and negative PNI	66 Gy in 33 fractions to the tumor bed	Ipsilateral neck (untreated); 10 months post-treatment	Alive 22 months post-treatment
70-year-old immune compromised man with cT3N1 SCC treated with definitive radiation and concurrent cemiplimab	66 Gy to gross disease in primary and lymph node and 60 Gy to the scalp and neck in 33 fractions	Contralateral neck (untreated); three months post-treatment	Died 5 months post-treatment

Abbreviations: SCC = squamous cell carcinoma, PNI = perineural invasion; V1 = 1st division of cranial nerve V
